# Diagnostic accuracy of linked administrative data for dementia diagnosis in community-dwelling older men in Australia

**DOI:** 10.1186/s12877-022-03579-2

**Published:** 2022-11-15

**Authors:** Eric P. F. Chow, Benjumin Hsu, Louise M. Waite, Fiona M. Blyth, David J. Handelsman, David G. Le Couteur, Vasi Naganathan, Fiona F. Stanaway

**Affiliations:** 1 Central Clinical School, Monash University, Melbourne, Victoria Australia; 2 Centre for Epidemiology and Biostatistics, Melbourne School of Population and Global Health, The University of Melbourne, Melbourne, Victoria Australia; 3 Melbourne Sexual Health Centre, Alfred Health, Melbourne, Victoria Australia; 4 Centre for Big Data Research in Health, UNSW Sydney, Sydney, New South Wales Australia; 5 School of Public Health, The University of Sydney, Sydney, New South Wales Australia; 6 Centre for Education and Research on Ageing, The University of Sydney and Concord Hospital, Sydney, New South Wales Australia; 7 ARC Centre of Excellence in Population Aging Research (CEPAR), The University of Sydney, Sydney, New South Wales Australia; 8 ANZAC Research Institute, The University of Sydney and Andrology, Concord Hospital, Sydney, New South Wales Australia; 9 Centre for Education and Research on Ageing, Faculty of Medicine and Health, The University of Sydney, Sydney, New South Wales Australia; 10 Ageing and Alzheimer’s Institute, Concord Repatriation and General Hospital, Sydney Local Health District, Sydney, New South Wales Australia; 11 Concord Clinical School, Faculty of Medicine and Health, The University of Sydney, Sydney, New South Wales Australia

**Keywords:** Medical records, Hospital records, Alzheimer’s disease, Sensitivity, Specificity, Predictive value

## Abstract

**Background:**

Routinely collected health administrative data can be used to estimate the prevalence or incidence of dementia at a population level but can be inaccurate. This study aimed to examine the accuracy of hospital and death data for diagnosing dementia compared with a clinical diagnosis in community dwelling older men in Australia.

**Methods:**

We performed a retrospective analysis of the Concord Health and Ageing in Men Project (CHAMP) in Sydney, Australia. Of the 1705 men aged ≥70 years in the CHAMP study, 1400 had available linked administrative data records from 1 year prior to 1 year post the date of clinical dementia diagnosis. The primary outcome was the accuracy of dementia diagnosis using linked administrative data records compared to clinical dementia diagnosis. The linked data diagnosis was based on hospital and death records for the 1 year pre and post the clinical diagnosis. Clinical dementia diagnosis was a two-stage process with initial screening, followed by clinical assessment for those meeting a validated cut-off. A final clinical diagnosis of dementia based on the Diagnostic and Statistical Manual of Mental Disorders (4th edition) criteria was reached by a consensus panel.

**Results:**

Administrative data identified 28 participants as having dementia, compared to 88 identified through clinical assessment. Administrative data had a sensitivity of 20% (95% CI: 13–30%, 18/88), specificity of 99% (95% CI: 99–100%, 1301/1312), positive predictive value (PPV) of 62% (95% CI: 44–77%), negative predictive value of 95% (95% CI: 94–95%), positive likelihood ratio of 24.4 (95% CI: 11.9–50.0) and negative likelihood ratio of 0.80 (0.72–0.89).

**Conclusions:**

Administrative hospital and death data has limited accuracy for dementia diagnosis with poor sensitivity and PPV. The prevalence of dementia is likely underestimated using hospital and deaths data.

**Supplementary Information:**

The online version contains supplementary material available at 10.1186/s12877-022-03579-2.

## Introduction

An estimated 472,000 people are living with dementia in Australia in 2021, [1] with 10% of people aged ≥65 years and 30% of people aged ≥85 years having dementia [2]. In Australia, the prevalence of dementia is generally estimated using a range of data sources including routinely collected health data such as hospitalisations, deaths and other health administrative data such as pharmaceutical claims for dementia-specific medications and aged care assessments [3–6]. However, the accuracy and agreement between these sources of data are not well understood. There are two important sources of inaccuracies in administrative data for dementia diagnosis. The first relates to inconsistent coding of dementia diagnosis from health records [7]. The second relates to the inability of such data to capture dementia diagnoses in those not using health services, including those who are not aware that they have dementia and thus have not sought a diagnosis. Furthermore, comorbidities (e.g. mild dementia) may not be identified in administrative data as these health conditions may not have had an impact on the episode of care and as a result would not be coded for in the hospital admission. This is an important consideration as it has been estimated that 62% of people living with dementia in the community are undiagnosed [8]. Moreover, this undercounting of dementia with administrative data may be more pronounced in individuals who have reduced access to or use of health services such as those with lower education or income and some ethnic minority groups [9–11].

Ideally, the population prevalence of dementia should be evaluated in a population-representative group using the gold standard diagnostic criteria which involves screening for cognitive impairment, followed by detailed clinical assessment on screen positive patients and a final diagnosis based on consensus by a panel of clinicians [12]. Depending on the effectiveness of the initial screening, such identification of people with dementia includes those that may have otherwise gone undiagnosed [8]. However, such comprehensive studies are resource intensive and not always feasible, particularly for large population-based cohorts [13]. Some countries have established a dementia registry with the potential to better understanding of the incidence of dementia. In 2020, Australia established the Australian Dementia Network (ADNeT) Registry but it will take several years for this registry to be useful for epidemiological monitoring [14]. In the absence of a registry, using routinely collected health administrative data, may be a cost-effective approach to determine the prevalence or incidence of dementia at a population level [3, 13, 15, 16].

This study aims to examine the diagnostic accuracy of hospital and deaths data for identifying individuals with dementia compared with a clinical dementia diagnosis in an ethnically diverse community-based cohort of men aged ≥70 years in Australia.

## Methods

### Study population

Data analysed come from the Concord Health and Ageing in Men Project (CHAMP): a cohort study of older men in Sydney, Australia. Further details of the methodology are described elsewhere [17]. In brief, 1705 men aged 70 years and over who lived in three local government areas (Burwood, Canada Bay and Strathfield) in inner western Sydney were recruited between 28 January 2005 and 4 June 2007. The New South Wales (NSW) Electoral Roll was used as the sampling frame for CHAMP which, as voting is compulsory in Australia, provides a representative and regularly updated sampling frame. The only exclusion criterion was living in a residential aged care facility. The baseline participation rate was 47% among eligible men with whom contact was made. Ethical approval was granted by the Sydney Local Health District Human Research Ethics Committee (HERC/14/CRGH/17). Written informed consent was obtained from all participants.

### Linked administrative data and dementia codes

Linked data on hospital records and deaths were obtained from the NSW Admitted Patient Data Collection (APDC) and the Australian Coordinating Registry (ACR) Cause of Death Unit Record File (COD-URF) from 1 July 2004 through the Centre for Health Record Linkage (CHeReL). The NSW APDC has data on the date of hospital admission and the assigned diagnosis codes using the International Statistical Classification of Diseases and Related Health Problems, Tenth Revision, Australian Modification (ICD-10-AM). Given there is no unique patient identifier in Australia, multiple records from the same patient are combined using a probabilistic record linkage approach [18]. The Australian Coordinating Registry (ACR) Cause of Death Unit Record File (COD UFR) provided cause of death data coded using International Classification of Diseases Tenth Revision (ICD-10).

There is no established validated time frame for assessing the diagnostic accuracy of administrative data for dementia diagnosis, and multiple different time frames have been used in past research ranging from 6 months to 2 years [19]. In this study, we defined a diagnosis of dementia using administrative data as having at least one hospital admission or death with a relevant ICD-10-AM code listed anywhere in the record in 1 year prior (hospital only) and 1 year post the date of the clinical dementia diagnosis (Table S1). This time frame was selected based on consultation with a geriatrician (LW) given the lack of a validated time frame [20]. All participants were successfully linked to the administrative databases. Participants with no hospital admission within the timeframe (474 men) were assumed to not have dementia as per standard practice [21–23].

### Reference standard

All CHAMP participants were screened for cognitive impairment using the Mini-Mental State Examination (MMSE) [24] in the baseline clinic visit. The 16-item Short Form of the Informant Questionnaire on Cognitive Decline in the Elderly (IQCODE) [25] was also completed at baseline by the nominated contact such as a spouse or child. Participants who did not have an IQCODE completed were not excluded. Participants with an MMSE score ≤ 26 and/or IQCODE score ≥ 3.6 were invited to undergo a detailed clinical assessment for dementia by a geriatrician. The geriatrician’s assessment lasted approximately 1–2 hours and included a review of medical comorbidities and medications, a standardized neurological assessment, a clinical dementia rating based on a detailed informant interview, [26] and the Rowland Universal Dementia Assessment Scale (RUDAS) in those whose first language was not English. RUDAS has been specifically designed for culturally and linguistically diverse populations and people with low education [27–29]. The final clinical diagnosis (i.e. normal, mild cognitive impairment, dementia, or unknown) was reached by consensus of a panel of two geriatricians, a neurologist and a neuropsychologist based on the Diagnostic and Statistical Manual of Mental Disorders (4th edition) criteria [30]. There were some men who met the criteria for further clinical assessment for dementia by a geriatrician where a diagnosis of dementia status was not reached. This was due to either (a) being unavailable to undergo further detailed assessment or (b) the panel being unable to reach a final consensus diagnosis based on the information available in those who had undergone further assessment. In both of these cases, such men were classified as having ‘unknown’ dementia status. The clinical diagnoses in CHAMP and dementia diagnoses reported in medical records were made blind to each other.

### Diagnosis of dementia based on administrative data

The primary outcome was based on administrative data records covering the 1 year prior (hospital data only) and the 1 year post the date of the clinical dementia diagnosis. The date of diagnosis was either the date of the baseline clinic assessment in those who screened negative based on the MMSE and IQCODE scores or was the date of the follow up clinical assessment in those who screened positive. In the primary analysis, we categorised individuals with a dementia status based on the clinical assessment as ‘unknown’ or ‘mild cognitive impairment’ as ‘no dementia’ for the diagnostic accuracy assessment.

### Statistical analyses

Descriptive statistics such as frequency, proportion, mean and standard deviation of the demographic characteristics were calculated and classified by clinically diagnosed dementia status (i.e. reference standard). The two-sample t-test was performed to compare the mean IQCODE between groups.

Six measures of diagnostic accuracy and their 95% confidence intervals (CI) were calculated to determine the accuracy of hospital and death records over a 2 year time frame (1 year pre and 1 year post the clinical diagnosis): sensitivity, specificity, positive predictive value (PPV), negative predictive value (NPV), positive likelihood ratio, and negative likelihood ratio [31].

Several sub-group and sensitivity analyses were performed in addition to the primary analysis. First, a sensitivity analysis for accuracy of diagnostic categories was undertaken by reclassifying the ‘unknown’ category of dementia diagnosis by (1) excluding all with an ‘unknown’ clinical diagnosis of dementia; or (2) grouping ‘unknown’ into the ‘dementia’ rather than ‘no dementia’ category. Second, different time frames for the administrative data records were used given the lack of an established and validated time frame in past research. The time frames were administrative data records covering (1) pre 6 months (hospital data only) and post 6 months; (2) only post 6 months; (3) only post 1 year; and (4) only post 2 years of the clinical dementia assessment. Both hospital and death data were used for the post-period. Third, we stratified the primary outcome by ethnicity using the following categories of the self-reported country of birth: (a) Australian-born (predominantly Anglo-Celtic background), (b) Italian or Greek migrants, and (c) all other migrant groups to test the hypothesis that accuracy of administrative data would vary between ethnic minority groups. Fourth, the accuracy of self-reported dementia in the CHAMP study was also compared to the reference standard, as well as combining self-reported dementia with pre and post 1 year linked administrative data.

All statistical analyses were performed in Stata (version 17, College Station, Texas, USA). This manuscript is reported as per the Standards for Reporting of Diagnostic Accuracy Studies (STARD) 2015 Guidelines (Table S2–3) [32].

## Results

**Fig. 1:**
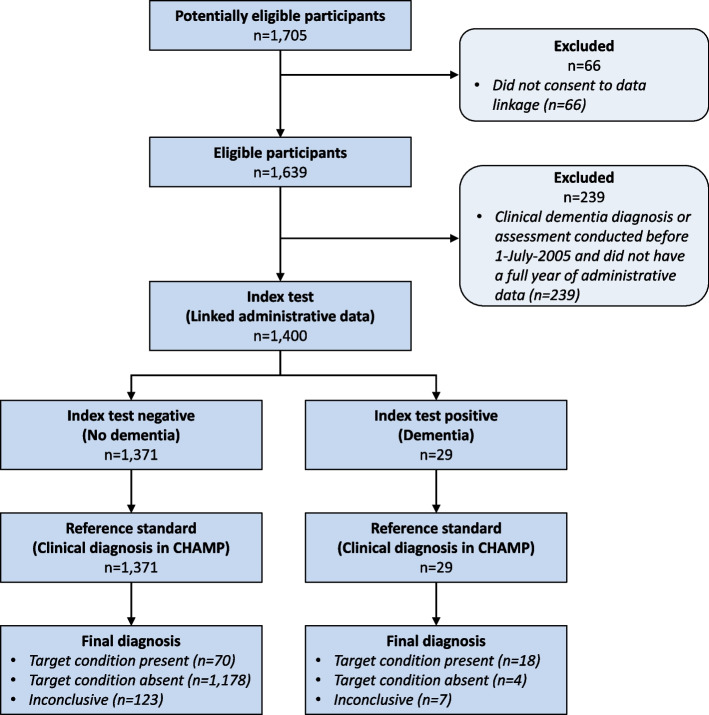
Flow chart of CHAMP recruitment and study subjects included in the present study

There were 1705 men recruited in the CHAMP at baseline, of whom 1639 (96%) consented to data linkage to administrative health records (Fig. 1). Linked data were available from 1 July 2004 to 4 May 2009 (i.e. 2 years after the date of the last enrolment in the CHAMP study). Therefore, participants who received their clinical dementia diagnosis or assessment before 1 July 2005 did not have a full year of linked administrative data available and were excluded from the primary analysis (i.e. pre and post 1 year linked administrative data). Of the 1400 men included in the primary analysis, 401 (29%) met the screening criteria and underwent additional assessment, 88 (6%) had a clinical diagnosis of dementia at baseline and 1312 (94%) men were categorised as having ‘no dementia’ (Table 1). Men without dementia were younger than men with dementia. Men without dementia were more often Australian-born than men with dementia (50% vs 40%) whereas the proportions of Italian or Greek migrants were similar but more other migrants were in the dementia group compared to the no dementia group (32% vs 26%). Men with dementia were more likely to live alone and less likely to have learnt English before the age of 12 years. Marital status was similar in both groups. Of the 88 men with dementia, 19 (22%) self-reported they had dementia, 67 (76%) self-reported no dementia and two (2%) were unsure. However, of the 1312 men without dementia, 18 (1%) self-reported that they had dementia.

**Table 1:** Demographic characteristics among 1400 men from CHAMP, by clinically diagnosed dementia status

Characteristic	No dementia (*N* = 1312)	Dementia (*N* = 88)
Age group, n (%)		
* 70–74*	531 (40.5%)	14 (15.9%)
* 75–79*	420 (32.0%)	21 (23.9%)
* 80–84*	235 (17.9%)	28 (31.8%)
* 85–89*	94 (7.2%)	17 (19.3%)
* 90–99*	32 (2.4%)	8 (9.1%)
Marital status, n (%)		
* Married or de facto*	1015 (77.4%)	67 (76.1%)
* Widowed, divorced or separated*	234 (17.8%)	17 (19.3%)
* Never married*	63 (4.8%)	4 (4.5%)
Country of birth, n (%)		
* Australia*	654 (49.8%)	35 (39.8%)
* Italy or Greece*	317 (24.2%)	25 (28.4%)
* Other*	341 (26.0%)	28 (31.8%)
Living alone, n (%)		
* Yes*	231 (17.6%)	21 (23.9%)
* No*	1073 (81.8%)	65 (73.9%)
*Unknown*	8 (0.6%)	2 (2.3%)
Language spoken at home, n (%)		
* English*	861 (65.6%)	54 (61.4%)
* Italian or Greek*	280 (21.3%)	21 (23.9%)
* Other*	171 (13.0%)	13 (14.8%)
Age for learning to speak English, n (%)		
*Before 12 years of age*	803 (61.2%)	48 (54.5%)
*After or equal to 12 years of age*	509 (38.8%)	40 (45.5%)
Self-reported dementia, n (%)		
* Yes*	18 (1.4%)	19 (21.6%)
* No*	1278 (97.4%)	67 (76.1%)
* Unsure*	16 (1.2%)	2 (2.3%)

**Fig. 2:**
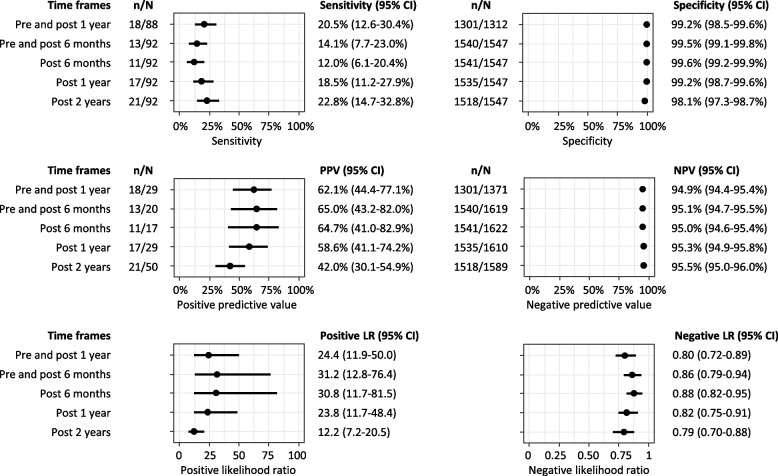
Sensitivity, specificity, positive predictive value, negative predictive value, positive likelihood ratio and negative likelihood ratio for administrative data for dementia diagnosis using different time frames compared to a clinical dementia diagnosis

There were 29 men with a dementia diagnosis using pre and post 1 year linked administrative data; of those 18 men had a clinical diagnosis of dementia, resulting in a PPV of 62% (95% CI: 44–77%) (Fig. 2, Table S4). Of the 88 men who had a clinical diagnosis of dementia, only 18 men had a dementia diagnosis from linked administrative data, giving a sensitivity of 20% (95% CI: 13–30%). The specificity of using pre and post 1 year linked administrative data was 99% (95% CI: 99–100%) and the NPV was 95% (95% CI: 94–95%). The mean IQCODE for the 18 true-positive cases was 4.3 (SD = 0.7), which was slightly higher than the 11 false-positive cases (3.8 [SD = 0.5], *p* = 0.024) and the 70 false-negative cases (3.7 [SD = 0.6], *p* < 0.001) but there was no difference in MMSE scores between groups. There were 474 (29%) men who did not have a hospitalisation or death record during the study period who were assumed to not have dementia. However, there was no significant difference in the mean number of hospital admissions among men who had a dementia record compared to men who did not have a dementia record (4.9 [SD 10.4] vs 2.2 [SD 11.3], *p* = 0.198).

The primary analysis shows that there were 11 false-positive cases identified as positive cases from the linked administrative data that were not identified as positive cases based on the clinical diagnosis we used as the reference standard: seven were men of unknown dementia status, four were men who were cognitively normal and none of them had mild cognitive impairment (Table S5). All seven men with unknown dementia status were screened positive for dementia based on MMSE (mean = 20.7) and IQCODE (mean = 4.0) but five of the men were unable to complete a follow up clinical assessment. If all 130 men with unknown dementia status were excluded, the PPV improved to 82% (95% CI: 61–93%) (Table S6, Fig. S1). Furthermore, if we assumed these 130 men with unknown dementia status had dementia, the PPV improved slightly further to 86% (95% CI: 69–95%) but the sensitivity dropped to 11% (95% CI: 8–16%) (Table S7, Fig. S2).

The sensitivity analyses using different time frames for the administrative data records were conducted among all 1639 men and the demographic characteristics of this larger sample were similar to the 1400 men in the primary analysis (Table S8). Figure 2 shows that sensitivity increased with a longer time frame for administrative data but remained low (ranging from 12% using post 6 months administrative data to 23% using post 2 years administrative data). The specificity remained high regardless of the time frame used (> 98%). The positive likelihood ratios for all time frames ranged from 12.2 to 31.2 and the negative likelihood ratios for all time frames ranged from 0.79 to 0.88.

Italian or Greek migrants had a lower sensitivity (12%; 95% CI: 3–31%) compared to the Australian-born (20%; 95% CI: 8–37%) and other migrants (29%; 95% CI: 13–50%) but the differences were not statistically significant with wide and overlapping confidence intervals (Table 2).

**Table 2:** Sensitivity, specificity, positive predictive value, negative predictive value, positive likelihood ratio and negative likelihood ratio for pre and post 1 year of administrative data for dementia diagnosis, stratified by country of birth

Test characteristics	Australia (*N* = 689)	Italy/Greece (N = 342)	Other (*N* = 369)
	*Result (95% CI)*	*Result (95% CI)*	*Result (95% CI)*
Sensitivity	20% (8–37%)	12% (3–31%)	29% (13–50%)
Specificity	99% (98–100%)	99% (98–100%)	99% (97–100%)
Positive predictive value	54% (29–78%)	60% (21–90%)	73% (43–90%)
Negative predictive value	96% (95–96%)	93% (93–94%)	94% (93–96%)
Positive likelihood ratio	21.8 (7.7–61.4)	19.0 (3.3–108.6)	32.5 (9.1–115.6)
Negative likelihood ratio	0.81 (0.68–0.95)	0.89 (0.77–1.02)	0.72 (0.57–0.91)

Note. Screening followed by clinical dementia diagnosis was used as the reference category

95% CI: 95% Confidence Interval

Adding self-reported dementia did not improve the diagnostic accuracy of the linked data diagnosis. There were small changes to the positive and negative likelihood ratios but not enough to change the clinical meaningfulness of the tests. For example, the negative likelihood ratio for no self-reported and no linked data diagnosis was not small enough to be useful in ruling out the presence of dementia (Tables S9–10).

## Discussion

Our findings suggest that routinely collected administrative hospital and death data has limited accuracy for dementia diagnosis. The poor sensitivity of 20% suggests that hospital and death data can only identify one-fifth of men with dementia, suggesting that the majority of men with dementia are not being captured in hospital or death data. Moreover, increasing the time frame of the administrative data does not greatly improve sensitivity. Hospital and death data did demonstrate relatively high specificity (> 98%) and NPV (> 95%). In addition, as the positive likelihood ratios for all time frames were all greater than 10, [33] a hospital and death data diagnosis is useful for ruling in a diagnosis of dementia. However, as the negative likelihood ratios for all time frames were greater than 0.1, [34] the lack of an administrative data diagnosis cannot rule out dementia.

Wilkinson et al. published a systematic review of 27 studies summarising the sensitivity of using routinely collected health data for identifying all-cause dementia [3]. However, most of the included studies with high sensitivity used medical chart review as the reference standard instead of clinical diagnosis. Using medical chart review as the reference standard to assess the accuracy of linked data is an inappropriate approach because the reference standard (medical chart review) is not independent of the index diagnostic test (dementia diagnosis codes) given that the dementia diagnosis codes are based on medical records. Moreover, medical chart review would not identify individuals with milder dementia who may not be screened or diagnosed yet and thus are not identified by administrative data.

In Wilkinson et al.’s review, only two studies used clinical diagnosis with a consensus panel of clinicians as a reference standard, which is similar to our current study [35, 36]. The estimated sensitivity in our study (21%) is similar to the estimate reported in the CAIDE (Cardiovascular Risk Factors, Aging and Dementia) in Finland using hospital discharge register records (14%) [35]; but it is lower than the estimate reported in the ADAMS (Aging, Demographics, and Memory Study) study in the US using Medicare claims (85%), which includes inpatient hospital claims, home health, and use of a skilled nursing facility. The inclusion of data on services outside of hospitals is likely behind the greater sensitivity observed in the ADAMS study. In Australia, in addition to hospital and death data, there are several other routine datasets that can be used to identify individuals with dementia, including pharmaceutical claims, aged care assessments, and the aged care funding instrument (ACFI) [4]. A previous Australian study used these five datasets to identify people with dementia and found that almost half of the people with dementia were identified in only one dataset [4]. The majority were from hospital records (32%), followed by ACFI (24%), pharmaceutical claims (23%), aged care assessments (17%) and death records (4%), [4] suggesting using multiple data sources would improve accuracy [13]. This is also supported by a Canadian study showing high sensitivity (79%) and PPV (80%) using an administrative data algorithm to identify dementia compared to electronic medical records by family physicians [37]. Unfortunately, we did not have access to these additional data sources in our study. However, it is still unlikely that such an algorithm would capture the same high number of people with dementia from screening and clinical diagnosis methods used in a community-based cohort study. In this design, it is possible to identify people who may be unaware of their dementia diagnosis and as a result not seeking health care.

The major strength of this study is that all men were screened for dementia with a final diagnosis based on clinical assessments and a consensus panel. The ethnic diversity of our sample is another strength as it is estimated that one in three Australians aged ≥65 years are migrants, with many born in a non-English speaking country [38]. We did not find strong evidence that ethnic background impacted the accuracy of dementia diagnosis using administrative data in our study. However, the small number of dementia cases in our community-based study meant that our comparison between ethnic groups was underpowered. Our study also has several limitations. First, we were only able to link hospital and death records, contributing to low sensitivity [39]. Second, we only included men in this study and the accuracy of using administrative data or self-reported dementia may be different between men and women [40]. Third, selection bias might have occurred in the CHAMP study due to the exclusion of those living in a residential aged care facility who would be more likely to have dementia [41]. Fourth, given the low prevalence of dementia (5.6%) in the community-based sample, we did not have sufficient power to examine whether the accuracy of administrative data varies according to demographic characteristics (e.g. socioeconomic status). Further studies using a larger sample size or an older cohort where dementia is more prevalent may be required. Fifth, MMSE scores can be influenced by culture, ethnicity and language, [42] which may cause bias in this ethnically diverse cohort. However, the final clinical diagnosis did use the results of additional assessments including the Rowland Universal Dementia Assessment Scale (RUDAS) in culturally and linguistically diverse men. Sixth, about 50% of the participants in our study were migrants. Our findings may be biased if these migrants are less likely to access healthcare services. Seventh, we might have underestimated the diagnostic accuracy of linked administrative data for dementia in this study as only health conditions affecting the episode of care are coded in APDC data and hence some men with dementia might not be identified.

To conclude, the sensitivity and PPV of linked administrative data are low using clinical diagnosis as the reference standard in an ethnically diverse community-based cohort of men. There has been increased use of administrative data in research, and estimates of the prevalence of dementia in Australia are based on administrative data given that there is limited cohort study data available. The services needed to provide support and care for men living with dementia are likely to be under-estimated if estimates are based on linked hospital and death data.

## Supplementary Information

**Additional file 1.**

## Data Availability

The datasets generated and/or analyzed during the current study are not publicly available due to ethical reasons but are available from the corresponding author on reasonable request.
