# Supplementary material for: Diagnostic accuracy of linked administrative data for dementia diagnosis in community-dwelling older men in Australia
Source: BMC Geriatr. 2022 Nov 15;22:858. doi: 10.1186/s12877-022-03579-2 (PMC9664614; doi:10.1186/s12877-022-03579-2)
Supplement: Supplementary file 1 — Additional file 1. [file 12877_2022_3579_MOESM1_ESM.docx]

**SUPPLEMENTARY MATERIALS**

**Table S1. A list of the ICD-10-AM code for dementia in linked data.**

| **ICD-10-AM code** | **Description** |
| --- | --- |
| F00 | Dementia in Alzheimer's disease |
| F01 | Vascular dementia |
| F02 | Dementia in other diseases classified elsewhere |
| F03 | Unspecified dementia |
| F05.1 | Delirium superimposed on dementia |
| G30 | Alzheimer's disease |
| G31.0 | Circumscribed brain atrophy |
| G31.1 | Senile degeneration of brain, not elsewhere classified |
| G31.2 | Degeneration of nervous system due to alcohol |
| G31.8 | Other specified degenerative diseases of nervous system |

**Table S2. Standards for Reporting of Diagnostic Accuracy Studies (STARD) 2015 Checklist**

| **Section & Topic** | **No.** | **Item** | **Reported on page #** |
| --- | --- | --- | --- |
| **TITLE OR ABSTRACT** |  |  |  |
|  | **1** | Identification as a study of diagnostic accuracy using at least one measure of accuracy  (such as sensitivity, specificity, predictive values, or AUC) | 1 |
| **ABSTRACT** |  |  |  |
|  | **2** | Structured summary of study design, methods, results, and conclusions  (for specific guidance, see STARD for Abstracts) | 1-2 |
| **Introduction** |  |  |  |
|  | **3** | Scientific and clinical background, including the intended use and clinical role of the index test | 3 |
|  | **4** | Study objectives and hypotheses | 4 |
| **METHODS** |  |  |  |
| Study design | **5** | Whether data collection was planned before the index test and reference standard were performed (prospective study) or after (retrospective study) | 4 |
| Participants | **6** | Eligibility criteria | 4 |
|  | **7** | On what basis potentially eligible participants were identified  (such as symptoms, results from previous tests, inclusion in registry) | 4 |
|  | **8** | Where and when potentially eligible participants were identified (setting, location and dates) | 4 |
|  | **9** | Whether participants formed a consecutive, random or convenience series | N/A |
| Test methods | **10a** | Index test, in sufficient detail to allow replication | 4-5 |
|  | **10b** | Reference standard, in sufficient detail to allow replication | 5-6 |
|  | **11** | Rationale for choosing the reference standard (if alternatives exist) | 5-6 |
|  | **12a** | Definition of and rationale for test positivity cut-offs or result categories of the index test, distinguishing pre-specified from exploratory | 4-6 |
|  | **12b** | Definition of and rationale for test positivity cut-offs or result categories of the reference standard, distinguishing pre-specified from exploratory | 4-6 |
|  | **13a** | Whether clinical information and reference standard results were available  to the performers/readers of the index test | 4-6 |
|  | **13b** | Whether clinical information and index test results were available  to the assessors of the reference standard | 5-6 |
| Analysis | **14** | Methods for estimating or comparing measures of diagnostic accuracy | 6-7 |
|  | **15** | How indeterminate index test or reference standard results were handled | 6-7 |
|  | **16** | How missing data on the index test and reference standard were handled | 6-7 |
|  | **17** | Any analyses of variability in diagnostic accuracy, distinguishing pre-specified from exploratory | 6-7 |
|  | **18** | Intended sample size and how it was determined | N/A |
| **RESULTS** |  |  |  |
| Participants | **19** | Flow of participants, using a diagram | Figure 1 |
|  | **20** | Baseline demographic and clinical characteristics of participants | Table 1 |
|  | **21a** | Distribution of severity of disease in those with the target condition | 7 |
|  | **21b** | Distribution of alternative diagnoses in those without the target condition | 7-8 |
|  | **22** | Time interval and any clinical interventions between index test and reference standard | 7 |
| Test results | **23** | Cross tabulation of the index test results (or their distribution)  by the results of the reference standard | 7-9, Table S5 |
|  | **24** | Estimates of diagnostic accuracy and their precision (such as 95% confidence intervals) | 7-9, Figure 2 |
|  | **25** | Any adverse events from performing the index test or the reference standard | N/A |
| **DISCUSSION** |  |  |  |
|  | **26** | Study limitations, including sources of potential bias, statistical uncertainty, and generalisability | 9-12 |
|  | **27** | Implications for practice, including the intended use and clinical role of the index test | 9-12 |
| **OTHER INFORMATION** |  |  |  |
|  | **28** | Registration number and name of registry | N/A |
|  | **29** | Where the full study protocol can be accessed | N/A |
|  | **30** | Sources of funding and other support; role of funders | 1 |

**Table S3. Standards for Reporting of Diagnostic Accuracy Studies (STARD) 2015 Checklist for Abstract: essential items for reporting diagnostic accuracy studies in journal or conference abstracts**

| **Section** | **Item** | **Reported on page #** |
| --- | --- | --- |
|  | Identification as a study of diagnostic accuracy using at least one measure of accuracy (such as sensitivity, specificity, predictive values, or AUC) | 1 |
| **Background and Objectives** | Study objectives | 1 |
| **Methods** | Data collection: whether this was a prospective or retrospective study | 1 |
|  | Eligibility criteria for participants and settings where the data were collected | 1 |
|  | Whether participants formed a consecutive, random, or convenience series | N/A |
|  | Description of the index test and reference standard | 1 |
| **Results** | Number of participants with and without the target condition included in the analysis | 1 |
|  | Estimates of diagnostic accuracy and their precision (such as 95% confidence intervals) | 1 |
| **Discussion** | General interpretation of the results | 2 |
|  | Implications for practice, including the intended use of the index test | 2 |

**Table S4. The 2🞨2 tables comparing dementia identified using linked administrative data to the reference standard of clinical diagnosis, stratified by different time frame for linked administrative data.** Men with mild cognitive impairment, unknown cognitive status and cognitively normal were categorised as ‘no dementia’.

| 1. **Pre and post one year*** | |  | |  |
| --- | --- | --- | --- | --- |
|  | | Reference standard (clinical diagnosis in CHAMP) | | Total |
|  |  | Dementia | No dementia |  |
| Linked administrate data | Dementia | 18 | 11 | 29 |
|  | No dementia | 70 | 1301 | 1371 |
| Total | | 88 | 1312 | 1400 |
| 1. **Pre and post 6 months** | |  | |  |
|  | | Reference standard (clinical diagnosis in CHAMP) | | Total |
|  |  | Dementia | No dementia |  |
| Linked administrate data | Dementia | 13 | 7 | 20 |
|  | No dementia | 79 | 1540 | 1619 |
| Total | | 92 | 1547 | 1639 |
| 1. **Post 6 months** | |  | |  |
|  | | Reference standard (clinical diagnosis in CHAMP) | | Total |
|  |  | Dementia | No dementia |  |
| Linked administrate data | Dementia | 11 | 6 | 17 |
|  | No dementia | 81 | 1541 | 1622 |
| Total | | 92 | 1547 | 1639 |
| 1. **Post 1 year** | |  | |  |
|  | | Reference standard (clinical diagnosis in CHAMP) | | Total |
|  |  | Dementia | No dementia |  |
| Linked administrate data | Dementia | 17 | 12 | 29 |
|  | No dementia | 75 | 1535 | 1610 |
| Total | | 92 | 1547 | 1639 |
| 1. **Post 2 years** | |  | |  |
|  | | Reference standard (clinical diagnosis in CHAMP) | | Total |
|  |  | Dementia | No dementia |  |
| Linked administrate data | Dementia | 21 | 29 | 50 |
|  | No dementia | 71 | 1518 | 1589 |
| Total | | 92 | 1547 | 1639 |

* Linked administrative data were available from 1 July 2004. Therefore, a total of 239 men who had their clinical dementia diagnosis or assessment before 1 July 2005 were excluded from the ‘pre and post one year’ analysis as these men did not have a full year of data before the clinical dementia diagnosis or assessment.

**Table S5. The number of individuals with and without dementia identified using pre and post one year of the administrative data, stratified by the final clinical diagnosis.**

|  | | Reference standard (clinical diagnosis in CHAMP) | | | | Total |
| --- | --- | --- | --- | --- | --- | --- |
|  |  | Dementia | MCI | Normal | Unknown |  |
| Linked administrative data | Dementia | 18 | 0 | 4 | 7 | 29 |
|  | No dementia | 70 | 107 | 1071 | 123 | 1371 |
| Total | | 88 | 107 | 1075 | 130 | 1400 |

Note. MCI = mild cognitive impairment.

**Table S6. The 2🞨2 tables comparing dementia identified using linked administrative data to the reference standard of clinical diagnosis, stratified by different time frame for linked administrative data.** Men with mild cognitive impairment and cognitively normal were categorised as ‘no dementia’ and 130 men with unknown cognitive status were excluded.

| 1. **Pre and post one year*** | |  | |  |
| --- | --- | --- | --- | --- |
|  | | Reference standard (clinical diagnosis in CHAMP) | | Total |
|  |  | Dementia | No dementia |  |
| Linked administrate data | Dementia | 18 | 4 | 22 |
|  | No dementia | 70 | 1178 | 1248 |
| Total | | 88 | 1182 | 1270 |
| 1. **Pre and post 6 months** | |  | |  |
|  | | Reference standard (clinical diagnosis in CHAMP) | | Total |
|  |  | Dementia | No dementia |  |
| Linked administrate data | Dementia | 13 | 3 | 16 |
|  | No dementia | 79 | 1394 | 1473 |
| Total | | 92 | 1397 | 1489 |
| 1. **Post 6 months** | |  | |  |
|  | | Reference standard (clinical diagnosis in CHAMP) | | Total |
|  |  | Dementia | No dementia |  |
| Linked administrate data | Dementia | 11 | 2 | 13 |
|  | No dementia | 81 | 1395 | 1476 |
| Total | | 92 | 1397 | 1489 |
| 1. **Post 1 year** | |  | |  |
|  | | Reference standard (clinical diagnosis in CHAMP) | | Total |
|  |  | Dementia | No dementia |  |
| Linked administrate data | Dementia | 17 | 5 | 22 |
|  | No dementia | 75 | 1392 | 1467 |
| Total | | 92 | 1397 | 1489 |
| 1. **Post 2 years** | |  | |  |
|  | | Reference standard (clinical diagnosis in CHAMP) | | Total |
|  |  | Dementia | No dementia |  |
| Linked administrate data | Dementia | 21 | 17 | 38 |
|  | No dementia | 71 | 1380 | 1451 |
| Total | | 92 | 1397 | 1489 |

* Linked administrative data were available from 1 July 2004. Therefore, a total of 369 men who had their clinical dementia diagnosis or assessment before 1 July 2005 or with an unknown cognitive status were excluded from the ‘pre and post one year’ analysis as these men did not have a full year of data before the clinical dementia diagnosis or assessment.

**Table S7. The 2🞨2 tables comparing dementia identified using linked administrative data to the reference standard of clinical diagnosis, stratified by different time frame for linked administrative data.** Men with mild cognitive impairment and cognitively normal were categorised as ‘no dementia’; while men with dementia and unknown cognitive status were categorised as ‘dementia’.

| 1. **Pre and post one year*** | |  | |  |
| --- | --- | --- | --- | --- |
|  | | Reference standard (clinical diagnosis in CHAMP) | | Total |
|  |  | Dementia | No dementia |  |
| Linked administrate data | Dementia | 25 | 4 | 29 |
|  | No dementia | 193 | 1178 | 1371 |
| Total | | 218 | 1182 | 1400 |
| 1. **Pre and post 6 months** | |  | |  |
|  | | Reference standard (clinical diagnosis in CHAMP) | | Total |
|  |  | Dementia | No dementia |  |
| Linked administrate data | Dementia | 17 | 3 | 20 |
|  | No dementia | 225 | 1394 | 1619 |
| Total | | 242 | 1397 | 1639 |
| 1. **Post 6 months** | |  | |  |
|  | | Reference standard (clinical diagnosis in CHAMP) | | Total |
|  |  | Dementia | No dementia |  |
| Linked administrate data | Dementia | 15 | 2 | 17 |
|  | No dementia | 227 | 1395 | 1622 |
| Total | | 242 | 1397 | 1639 |
| 1. **Post 1 year** | |  | |  |
|  | | Reference standard (clinical diagnosis in CHAMP) | | Total |
|  |  | Dementia | No dementia |  |
| Linked administrate data | Dementia | 24 | 5 | 29 |
|  | No dementia | 218 | 1392 | 1610 |
| Total | | 242 | 1397 | 1639 |
| 1. **Post 2 years** | |  | |  |
|  | | Reference standard (clinical diagnosis in CHAMP) | | Total |
|  |  | Dementia | No dementia |  |
| Linked administrate data | Dementia | 33 | 17 | 50 |
|  | No dementia | 209 | 1380 | 1589 |
| Total | | 242 | 1397 | 1639 |

* Linked administrative data were available from 1 July 2004. Therefore, a total of 239 men who had their clinical dementia diagnosis or assessment before 1 July 2005 or with an unknown cognitive status were excluded from the ‘pre and post one year’ analysis as these men did not have a full year of data before the clinical dementia diagnosis or assessment.

**Table S8. Demographic characteristics among 1639 men from CHAMP, by clinically diagnosed dementia status.**

| **Characteristic** | **No dementia (N=1547)** | **Dementia (N=92)** |
| --- | --- | --- |
| Age group, n (%) |  |  |
| 70-74 | 629 (40.7%) | 14 (15.2%) |
| 75-79 | 492 (31.8%) | 24 (26.1%) |
| 80-84 | 274 (17.7%) | 29 (31.5%) |
| 85-89 | 114 (7.4%) | 17 (18.5%) |
| 90-99 | 38 (2.5%) | 8 (8.7%) |
| Marital status, n (%) |  |  |
| Married or de facto | 1186 (76.7%) | 70 (76.1%) |
| Widowed, divorced or separated | 282 (18.2%) | 18 (19.6%) |
| Never married | 79 (5.1%) | 4 (4.3%) |
| Country of birth, n (%) |  |  |
| Australian | 793 (51.3%) | 36 (39.1%) |
| Italian or Greek migrants | 357 (23.1%) | 27 (29.3%) |
| Other migrants | 397 (25.7%) | 29 (31.5%) |
| Living alone |  |  |
| Yes | 285 (18.4%) | 22 (23.9%) |
| No | 1253 (81.0%) | 68 (73.9%) |
| Unknown | 9 (0.6%) | 2 (2.2%) |
| Language spoken at home |  |  |
| English | 1045 (67.6%) | 57 (62.0%) |
| Italian or Greek | 312 (20.2%) | 22 (23.9%) |
| Other | 190 (12.3%) | 13 (14.1%) |
| Age for learning to speak English, n (%) |  |  |
| Before 12 years of age | 974 (63.0%) | 51 (55.4%) |
| After or equal to 12 years of age | 573 (37.0%) | 41 (44.6%) |
| Self-reported dementia |  |  |
| Yes | 20 (1.3%) | 21 (22.8%) |
| No | 1508 (97.5%) | 69 (75.0%) |
| Unsure | 19 (1.2%) | 2 (2.2%) |

**Table S9. The likelihood ratio of individuals who self-reported dementia, no dementia and unsure compared to the reference standard of clinical diagnosis.**

|  | | Reference standard  (clinical diagnosis in CHAMP) | | Total | Likelihood ratio |
| --- | --- | --- | --- | --- | --- |
|  |  | Dementia | No dementia* |  |  |
| Self-reported | Dementia | 21 | 20 | 41 | 17.7 (9.9-31.4) |
|  | No dementia | 69 | 1508 | 1577 | 0.77 (0.68-0.87) |
|  | Unsure | 2 | 19 | 21 | 1.8 (0.4-7.5) |
| Total | | 92 | 1547 | 1639 | - |

* Men with mild cognitive impairment, unknown cognitive status and cognitively normal were categorised as ‘no dementia’.

**Table S10. The likelihood ratio of individuals who self-reported dementia, no dementia and unsure in addition to pre and post one year of administrative data, compared to the reference standard of clinical diagnosis.**

|  | | Reference standard (clinical diagnosis in CHAMP) | | Total | Likelihood ratio |
| --- | --- | --- | --- | --- | --- |
|  |  | Dementia | No dementia* |  |  |
| Self-reported & linked administrative data | Dementia | 29 | 27 | 56 | 18.1 (11.2-29.2) |
|  | No dementia | 61 | 1502 | 1563 | 0.68 (0.59-0.79) |
|  | Unsure | 2 | 18 | 20 | 1.9 (0.44-7.9) |
| Total | | 92 | 1547 | 1639 | - |

* Men with mild cognitive impairment, unknown cognitive status and cognitively normal were categorised as ‘no dementia’.

**Figure S1. Sensitivity, specificity, positive predictive value, negative predictive value, positive likelihood ratio and negative likelihood ratio for administrative data for dementia diagnosis using different time frames compared to a clinical dementia diagnosis.** Men with mild cognitive impairment and cognitively normal were categorised as ‘no dementia’ and 150 men with unknown cognitive status were excluded.

**Figure S2. Sensitivity, specificity, positive predictive value, negative predictive value, positive likelihood ratio and negative likelihood ratio for administrative data for dementia diagnosis using different time frames compared to a clinical dementia diagnosis.** Men with mild cognitive impairment and cognitively normal were categorised as ‘no dementia’; while men with dementia and unknown cognitive status were categorised as ‘dementia’.
